# Interference with the *C*-terminal structure of MARF1 causes defective oocyte meiotic division and female infertility in mice

**DOI:** 10.7555/JBR.32.20170108

**Published:** 2018-01-26

**Authors:** Guang-Yi Cao, Ming-Zhe Li, Hao Wang, Lan-Ying Shi, You-Qiang Su

**Affiliations:** 1. State Key Laboratory of Reproductive Medicine, Nanjing Medical University, Nanjing, Jiangsu 211166, China; 2. The Third Affiliated Hospital, Guangzhou Medical University, Guangzhou, Guangdong 510150, China; 3. Collaborative Innovation Center of Genetics and Development, Fudan University, Shanghai 200433, China; 4. Key Laboratory of Model Animal Research, Nanjing Medical University, Nanjing, Jiangsu 211166, China.

**Keywords:** MARF1, meiosis, oocyte aneuploidy, female infertility, knock in, CRISPER/Cas9

## Abstract

Meiosis-arrest female 1 (MARF1) is a recently identified key oogenic regulator essential for the maintenance of female fertility and genome integrity in mice. However, the detailed functions and the underlying mechanisms of MARF1 remain elusive. Here, in an attempt to create a mouse model expressing fluorescent protein-tagged MARF1 to facilitate further exploration of the roles of MARF1 in oocytes, we produced a *Marf1-eGFP* knockin (KI) mouse line in which the *C*-terminal structure and function of MARF1 were interfered by its fusing eGFP peptide. Using these *Marf1-eGFP*-KI mice, we revealed, unexpectedly, the functions of MARF1 in the control of oocyte meiotic division. We found that the *Marf1-eGFP*-KI females ovulated mature oocytes with severe meiotic and developmental defects, and thus were infertile. Moreover, meiotic reinitiation was delayed while meiotic completion was accelerated in the KI-oocytes, which was coincident with the increased incidence of oocyte aneuploidy. Therefore, MARF1 is indispensable for maintaining the fidelity of homolog segregation during oocyte maturation, and this function relies on its *C*-terminal domains.

## Introduction

The success of reproduction necessitates the production of functionally normal eggs. Oocyte maturation is an essential step in producing a high-quality haploid egg which, upon fertilization, is competent to produce a diploid embryo and eventually gives rise to a healthy new individual. To achieve this, precise coordination between the inner cell cycle progression and the developmental program is required^[1]^. Fully grown oocytes of most mammalian species are arrested at the diplotene stage of the first meiotic prophase, which is most analogous to the G2 phase of the mitotic cell cycle division. At this stage, the oocyte has a large intact nucleus termed germinal vesicle (GV). The resumption of meiosis, equivalent to the mitotic G2/M transition^[2]^, only takes place under the stimulation of the preovulatory LH surge or removal of oocytes from their follicular environment. This process, commonly referred to as GV breakdown (GVB), is manifested by the dissolution of the nuclear envelop and disappearance of the nucleolus. Following GVB, microtubules nucleate to form meiotic spindles and chromosomes undergo condensation. The first meiosis then proceeds through pro-metaphase I (pro-MI), metaphase I (MI) and anaphase-telophase I (AI-TI). Upon the completion of the TI, associated with the segregation of the homologous chromosomes, the oocyte extrudes the first polar body (PB1), and meiosis directly reaches metaphase II (MII) without the intervening interphase. Meiosis is again arrested at MII, and will not resume until sperm-induced oocyte activation occurs.

Meiosis's precision in timing and accuracy of chromosome segregation are critical for ensuring the quality of the resulting eggs, and determine the fate of the future embryos after fertilization. Meiotic defects can result in errors in chromosome segregation, which are the leading causes of aneuploidy. Oocyte aneuploidy is the predominant genetic causes of human infertility, miscarriage and birth defects, and is also the major factor that restrains the success of assistant reproductive technology (ART) in clinics. As human oocytes can be arrested for decades in prophase I, their first meiotic division is particularly error prone. Indeed, errors of the first meiosis account in a large part for the unusual high aneuploidy rate of the fertilized eggs, which presents a basal rate close to 20% in women younger than 35 years of age, and increases even up to 60% with advancing maternal age^[3]^. Therefore, studying the molecular control of oocyte meiotic division is of great importance in helping unravel the mechanisms underlying such severe incidence of human aneuploidies. Though amazing progress has been made, much still needs to learn^[3– 7]^.

We have been using a forward genetics approach–"ENU-induced mouse mutagenesis" to screen genes that are essential for oogenesis. Excitingly, we have identified recently a novel Riken gene that is robustly expressed by oocytes and functions in the control of both meiotic progression and retrotransposon silencing in mouse oocytes. Mutations of this gene cause female only infertile phenotype owing to meiotic arrest and ovulation of immature GV-stage oocytes; we therefore named this gene meiosis arrest female 1, *Marf1*. The function of *Marf1*'s orthologs and/or homologs in any other model organisms has not yet been appreciated^[8– 9]^. A deeper and extensive investigation of the function of MARF1 in the control of oogenesis and the underlying mechanisms is therefore very necessary.

In the present study, we aim to create a mouse model expressing fluorescent protein-tagged MARF1, and to use this model to further explore the roles of MARF1 in oocytes. To our surprise, we serendipitously produced a *Marf1-eGFP* knockin (KI) mouse line, in which the *C*-terminal structure and function of the expressed MARF1 protein were interfered by its fusing eGFP peptide. By studying this line of mice, we unexpectedly revealed the indispensable role of MARF1 in the control of oocyte meiotic division.

## Materials and methods

### Animals

All mice were bred and raised in the researcher's colony at Nanjing Medical University Animal Core Facility, a standard animal facility accredited by the Chinese Association for Assessment and Accreditation of Laboratory Animal Care. All experiments and animal procedures were approved by the Animal Care and Use Committee of Nanjing Medical University, and were performed in accordance with institutional guidelines.

### Production of Cas9 mRNA, sgRNA and donor DNA

To produce the template for in vitro synthesis of Cas9 mRNA, T7 promoter was inserted into the PX330 vector, with its position sitting just in front of the Cas9 coding sequence. The fragment harboring T7-Cas9 sequences was then amplified by PCR using the primers specific for T7-Cas9^[10– 11]^. The amplified T7-Cas9 PCR products were then collected and purified using the QIAquick Gel Extraction Kit (Qiagen, USA). After purification, 500 ng of it was picked out for in vitro transcription using the mMESSAGE mMACHINE T7 ultra transcription kit (Ambion, USA). Specific sgRNAs targeting the 3′- end of the last exon of *Marf1* gene were designed using the online tool for design of sgRNAs (http://crispr.mit.edu/). The template for *in vitro* synthesis of sgRNA was produced using the same procedure as producing Cas9, and 250 ng of the purified template was used for *in vitro* transcription using MEGA short script T7 kit (Ambion, USA). Both Cas9 mRNA and specific sgRNA were purified according to the standard protocol by phenol:chloroform extraction and ethanol precipitation, and then dissolved in DNase/RNase-free water (Life Technologies, USA). Primer sequences are listed in ***Supplementary Table 1***, available online. Donor DNA was constructed by In-Fusion® HD Cloning Kit (Clontech, Japan). Gene-specific donor DNA for HDR (homology directed repair) is listed in ***Supplementary Table 2***, available online.

### Generation of knockin mice expressing MARF1-eGFP fusion protein

Zygotes were collected from (B6xDBA2) F1 (hereafter referred to as BD2F1) female mice that were mated with BD2F1 males after superovulation treatment. The mixture of Cas9 mRNA (100 ng/µL), sgRNA (50 ng/µL), and donor DNA (10–20 ng/µL) were then injected into the cytoplasm of the resultant zygotes in CZB (Chatot-Ziomek-Bavister) medium. After culturing the injected zygotes for 24 hours to let them develop into the 2-cell stage embryos, 18-20 of them were transferred into the oviduct of a surrogate pseudopregnant ICR female mouse that was prepared by mating with a vasectomized male.

### Oocyte collection and culture

Fully grown oocytes arrested at the germinal vesicle (GV)-stage were harvested from the ovaries of 3-week-old females 46-48 hours after the initial priming with 5 IU of pregnant mare serum gonadotropin, PMSG (Ningbo Second Hormone Factory, China) to stimulate antral follicle development. Cumulus-oocyte complexes (COCs) were collected and the surrounding cumulus cells were removed by repeatedly pipetting the complexes. All the process was carried out in MEM-alpha supplemented with 3 mg/mL of bovine serum albumin (BSA, Sigma, USA) and 5 mmol/L Milrinone (Calbiochem, Germany) to maintain the oocyte at GV stage. For in vitro maturation, GV-stage oocytes were incubated in MEM (Gibco, USA) supplemented with 75 mg/mL penicillin G (Sigma), 50 mg/mL streptomycin sulfate (Sigma), 25 mg/mL pyruvate (Sigma), 38 mg/mL EDTA (Sigma) and 3 mg/mL BSA. Culture condition was maintained at 37°C in an incubator infused with 5%O_2_, 5%CO_2_ and 90%N_2_. Oocytes at MII stage were collected from oviductal ampullae of the female mice 14-16 hours after they were received the superovulation treatment. Superovulation was induced by intraperitoneal injection of the PMSG-primed (46–48 hours) mice with 5 IU human chorionic gonadotropin (hCG, Ningbo Second Hormone Factory, China).

### Immunofluorescence

Mouse oocytes were fixed with 4% paraformaldehyde (PFA) for 45 minutes at room temperature, then blocked in the blocking buffer (10% BSA and 1% Triton X-100 in PBS) for 1 hour at room temperature. After blocking, the oocytes were incubated overnight at 4°C with FITC-conjugated anti-α-tubulin antibody (1:500, Millipore-Sigma, USA). After washing 3 times, the oocytes were then incubated with Texas Red-conjugated phalloidin (1:750, Invitrogen; USA) for 1 hour at room temperature. Chromosomes were counter stained with Hoechst 33342 for 15 minutes. The oocytes were mounted on glass slides and examined under a laser scanning confocal microscope (LSM 700, Carl Zeiss, Germany).

### Western blotting assays

Eighty mouse oocytes were collected in sample buffer and boiled for 5 minutes at 110°C. Denatured proteins were separated by 7.5% SDS-PAGE and transferred to PVDF membrane. For blocking, membranes were immersed in PBS with 0.1% Tween-20 (PBST) and 5% low-fat dry milk for 1 hour at room temperature, followed by incubation at 4°C overnight with the rabbit anti-MARF1 (1:2,000, produced in house) and mouse anti–β-actin (1:2,000, Sigma) antibodies. Subsequently, membranes were incubated with HRP-conjugated secondary antibodies in blocking buffer for 1 hour after washing 3 times in PBST. Finally, membranes were washed 3 times in PBST and then visualized by using an ECL Plus Western Blotting Detection System (GE Healthcare, USA).

### Chromosome spread

Zona pellucida was removed by exposing oocytes in Tyrode's buffer (pH2.5) for 30 seconds at 37°C. Subsequently, the oocytes were fixed in a drop of 1% PFA with 0.15% Triton X-100 on a glass slide. The oocytes were air-dried, and incubated with the human anti-centromere antibodies (1:500, Antibodies Incorporated, USA) at 4°C overnight, followed by incubating with donkey anti-Human IgG Alexa Fluor594-conjugated secondary antibodies. Then, the chromosomes were counter stained with Hochest 33342. The specimens were examined under a laser scanning confocal microscope (LSM 700, Carl Zeiss, Germany).

### ***In vitro*** fertilization

Oocytes at MII stage were harvested from the oviducts of 24-day-old female mice that were initially received superovulation treatment. Sperms were collected from caudal epididymides of 10-week-old B6D2F1 male mice with normal fertility, and were capacitated for 1 hour at 37°C in an incubator infused with 5%O_2_, 5%CO_2_ and 90%N_2_. *In vitro* fertilization (IVF) was then carried out as described previously^[12]^. The formation of 2-cell and blastocyst-stage embryos was assessed 24 hours and 96 hours after IVF.

### Statistical analysis

Data are presented as mean±SEM, unless otherwise indicated. Differences between 2 groups were analyzed by Student's *t*-test. Multiple comparisons between more than 2 groups were analyzed by one-way ANOVA followed by Tukey's honest significant difference (HSD) test using Prism 5.0. *P*≤0.05 was considered to be significantly different.

## Results

### Generation of knockin mice expressing MARF1-eGFP fusion protein by CRISPR/Cas9-mediated genome editing

**Fig.1:**
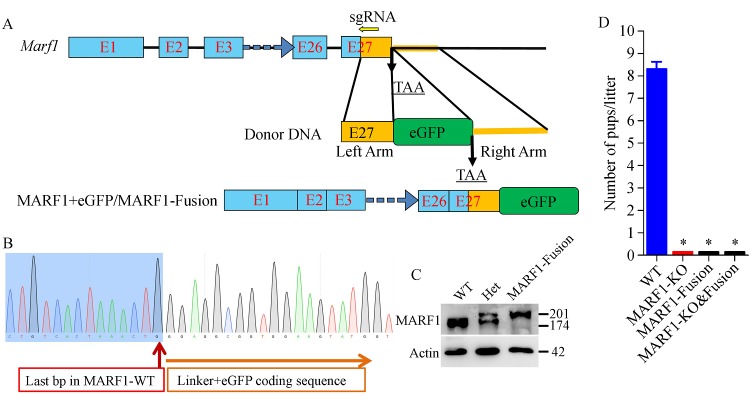
CRISPR/Cas9-mediated knockin of ***eGFP*** at the C-terminus of MARF1 causes female infertility.

To facilitate further exploration of the role of MARF1 in oocytes, especially its dynamic expression and localization during oocyte development, we sought to create a mouse model that expressed fluorescent protein-tagged MARF1 in oocytes. CRISPR/Cas9-mediated knockin of eGFP at the C-terminus of MARF1 was carried out by microinjecting the specific sgRNA, high-efficient Cas9 mRNA, and eGFP donor DNA into zygotes, followed by transfer of the injected embryos into the surrogate pseudopregnant female mice **(*****Fig. 1A*****)**. As indicated by the genotyping sequencing result **(*****Fig. 1B, Supplementary Fig. 1***, available online **)**, the eGFP cassette was successfully integrated to the 3′-end of the last exon of *Marf1* right before the stop codon. Western blot analysis showed that the oocytes of the mice carrying this knockin allele expressed the MARF1-eGFP fusion protein **(*****Fig. 1C*****)**. The molecular weight of MARF1 and eGFP protein was 174kDa and 27kDa, respectively. As expected, a single 174kDa band was detected in the sample of wild type (WT) oocytes, and a single larger band with the size of approximately 201 kDa (equal to 174 kDa MARF1 plus 27 kDa eGFP) was detected in the sample of *Marf1*-eGFP-KI (MARF1-Fusion) oocytes. Two bands, with the sizes of 174 and 201 kDa, respectively, were detected in the sample of heterozygous KI oocytes **(*****Fig. 1C*****)**.

### Fusion with eGFP interferes with the C-terminal structure and function of MARF1 and causes female infertility

One of the original purposes of creating the KI-mice is to track the dynamic localization and expression of MARF1 protein during oocyte development. However, no eGFP signal was detected either by direct live imaging of the oocytes isolated from the KI-mice under fluorescent microscope, or by indirect immunostaining of the ovarian sections and isolated oocytes of the KI-mice using the anti-eGFP antibody (data not shown). These data suggest that the eGFP peptide fusing to the C terminus of MARF1 is probably masked during the MARF1's posttranslational processing and folding.

To our surprise, we found unexpectedly that the *Marf1*-eGFP-KI (MARF1-Fusion) mice displayed a female-only infertile phenotype as did by the *Marf1*gene trap (MARF1-KO) mice, with no pups produced during the whole period of fertility test **(*****Fig. 1D*****)**. Allelic complementation test demonstrated that the females carrying both the *Marf1* gene trap allele and the *Marf1*-eGFP-KI allele (MARF1-KO&Fusion) were also completely infertile **(*****Fig. 1D*****)**, thus indicating that the infertile phenotype was attributable to the defect of MARF1.

### ***Marf1***-eGFP-KI female mice ovulate low-quality eggs

**Fig.2:**
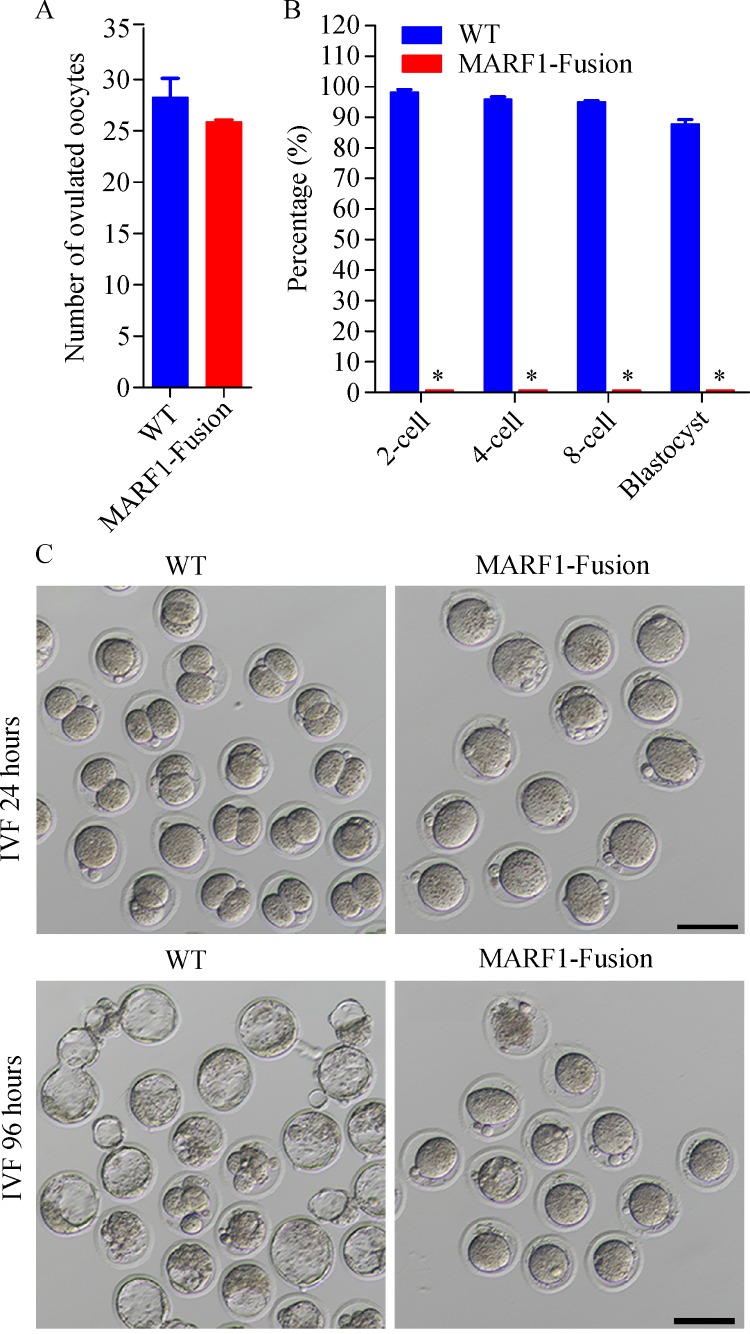
Compromised competence of development in eggs ovulated by ***Marf1***-eGFP knockin mice.

To figure out what causes the *Marf1*-eGFP-KI females infertility, ovulation performance and the quality of the ovulated eggs were assessed. As shown in ***Fig. 2A***, *Marf1*-eGFP-KI females ovulated similar number of eggs as WT controls. However, when these eggs were inseminated *in vitro* with normal WT sperms, no normal 2-cell stage embryos were formed, even after culture for extended days when the WTs have formed blastocysts **(*****Fig. 2B-D*****)**. These data indicate that although the *Marf1*-eGFP-KI females can ovulate mature eggs, the ovulated eggs in essence are developmentally incompetent.

**Fig.3:**
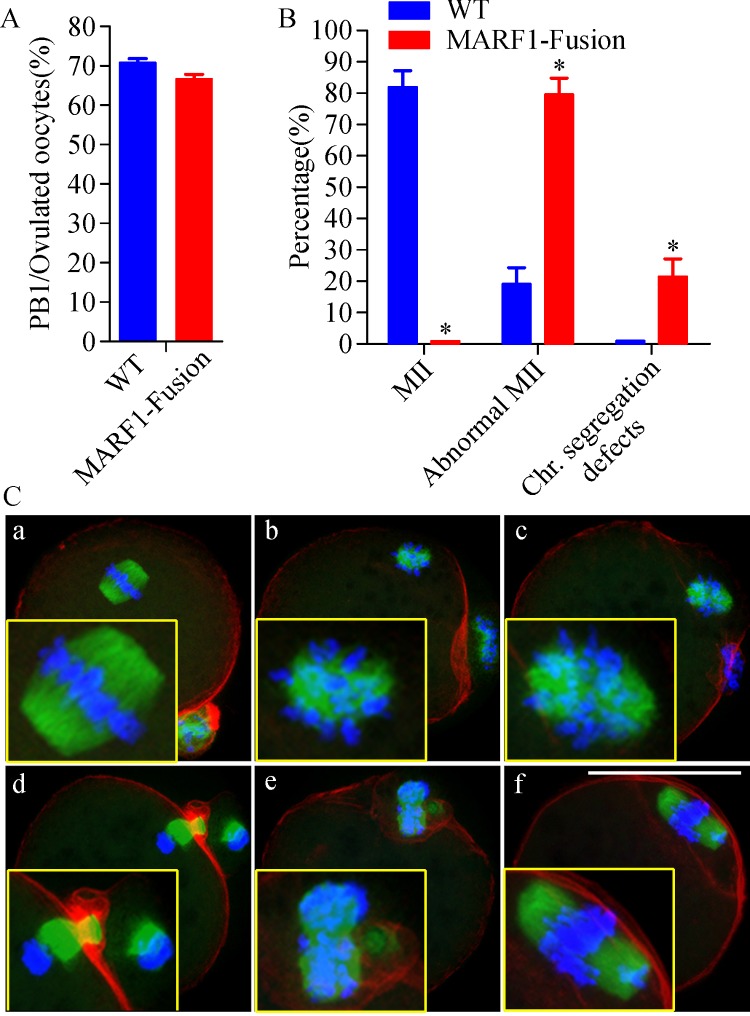
Defective meiotic progression to MII in the ovulated oocytes of ***Marf1***-eGFP knockin mice.

Because meiotic defects are major contributors to reduced egg quality^[3]^, we examined if the first meiosis was completed normally in the *Marf1*-eGFP-KI eggs. The meiotic status of the ovulated eggs was evaluated by confocal microscopy after immunofluorescent staining of the meiotic spindles, chromosomes and F-actins. As shown in ***Fig. 3A***, there was no difference in the rate of the first polar body (PB1) extrusion between WT and *Marf1*-eGFP-KI eggs. However, the incidence of meiotic defects was extremely high in the *Marf1*-eGFP-KI eggs as compared with that of the WTs, with nearly none of the *Marf1*-eGFP-KI eggs reached MII properly and formed normal spindles **(*****Fig. 3B*****)**. The majority of these *Marf1*-eGFP-KI eggs formed abnormal MII spindles with misaligned chromosomes scattered around **(*****Fig. 3A, C-b,c*****)**. A significant proportion of them displayed defective cytokinesis and/or chromosome separation, with the homologous chromosomes separated incompletely **(*****Fig. 3A, C-d,e*****)**, or all the homolog went into the polar body **(*****Fig. 3A, C-f*****)**. Therefore, the completion of the first meiosis was severely compromised in the ovulated *Marf1*-eGFP-KI eggs.

### The kinetics of the progression of the first meiosis is skewed in ***Marf1***-eGFP-KI oocytes

**Fig.4:**
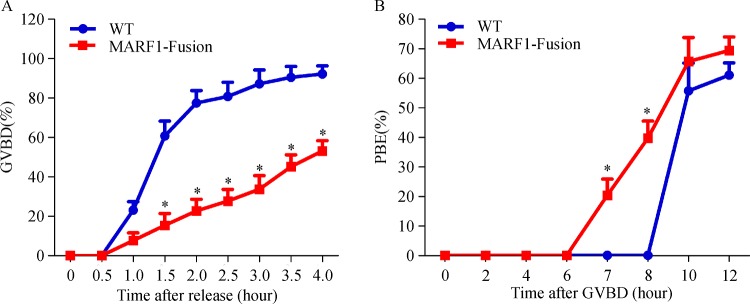
Kinetics of the progression of the first meiosis in WT and ***Marf1***-eGFP-KI oocytes.

The defects in completion of the first meiosis observed in the ovulated *Marf1*-eGFP-KI eggs suggest that meiotic progression in *Marf1*-eGFP-KI oocytes is probably also compromised. We therefore examined the kinetics of *Marf1*-eGFP-KI oocyte meiotic progression by maturating them in culture. We found that both the resumption and the completion of the first meiosis, as manifested by GVB and the PB1 extrusion, respectively, took place in a normal kinetics in the WT oocytes. Most (80%) of the WT oocytes completed GVB within 2 hours, and 60% percent of them extruded PB1 10 hours after GVB **(*****Fig. 4A, B*****)**. However, the kinetics of meiotic progression in the *Marf1*-eGFP-KI oocytes was severely skewed. GVB was significantly delayed in the *Marf1*-eGFP-KI oocytes, with less than 25% of them restored meiosis within 2 hours, and only 50% completed GVB 4 hours after culture **(*****Fig. 4A*****)**. Nevertheless, the extrusion of PB1 was, however, accelerated in the *Marf1*-eGFP-KI oocytes. It started 7 hours after GVB, and already reached 40% by 8 hours after GVB occurred **(*****Fig. 4B*****)**.

### Distortion of the spindle apparatus and increased errors in chromosome alignment and segregation in ***Marf1***-eGFP-KI oocytes

**Fig.5:**
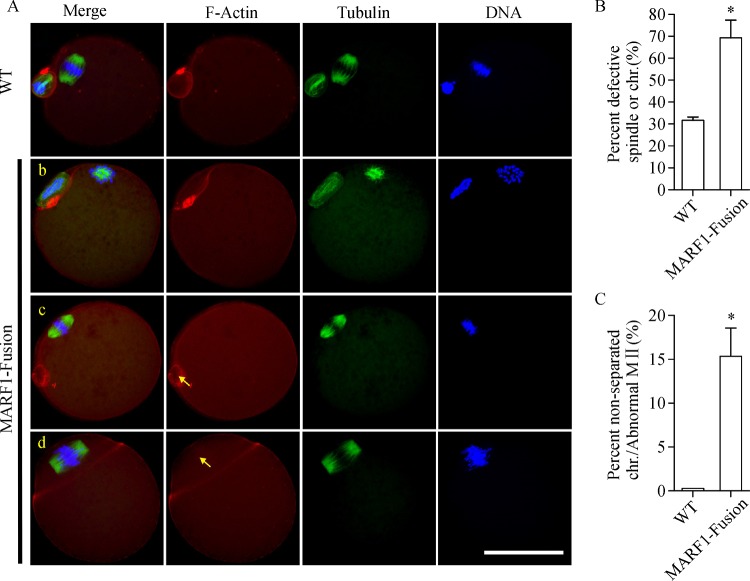
Defective progression of meiosis to metaphase II by ***Marf1***-eGFP-KI oocytes ***in vitro***.

**Fig.6:**
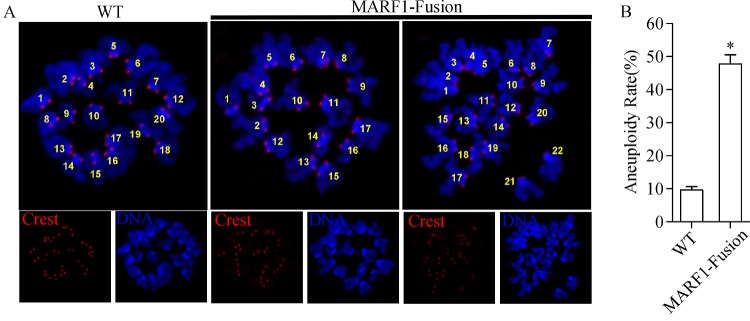
Increased incidence of aneuploidy in ***Marf1***-eGFP-KI oocytes after maturation ***in vitro***.

The unusual kinetics of meiotic progression in *Marf1*-eGFP-KI oocytes, especially the earlier onset of PB1 extrusion, suggests that the meiotic spindle apparatus and the dynamics of chromosomes are probably also skewed. Indeed, we found that after maturation *in vitro*, there were more *Marf1*-eGFP-KI oocytes displaying distorted meiotic spindles and misaligned chromosomes, which was about 2-fold more of WTs **(*****Fig. 5A, B*****)**. Most of these oocytes had completed the first meiotic division, with their homologous chromosomes separated **(*****Fig. 5A-b*****)**. However, there was a significant proportion (15%) of the abnormal oocytes having homologous nondisjunction, with the whole sets of chromosome along with the associated MI spindles either retained in the oocyte or moved into the extruded PB1 after the cytogenesis **(*****Fig. 5A-c, d, and C*****)**. Consistent with these abnormalities in spindles and chromosomes, the incidence of aneuploidy was remarkably high in the *Marf1*-eGFP-KI mature oocytes, with the rate increased about 4-fold more than that of the WT oocytes **(*****Fig. 6 A, B*****)**.

## Discussion

Egg quality is a key limiting factor for the success of reproduction, and determines the outcome of pregnancy and the health status of the resulting individual. To produce a "good" egg, the oocyte must resume and complete the first meiosis. Meiosis's precision in timing and the fidelity of homolog segregation are thus crucial for ensuring the egg quality. MARF1 is a recently identified key oogenic regulator that specifically controls the resumption of meiosis in oocytes^[89]^. Here, we revealed that MARF1 was also indispensable for oocyte completion of the first meiosis. Interruption of the normal structure and function of its C-terminal domains causes multiple defects in oocyte meiosis, which include the skewed kinetics of meiotic progression, distorted spindle apparatus and the chromosome dynamics, and the marked elevation of aneuploidy. These meiotic defects compromise the quality of the ovulated eggs, and eventually lead to infertility in the female mice. MARF1 is therefore a novel regulator of oocyte meiotic maturation.

MARF1 is annotated to be a RNA binding protein. It contains 3 major domains: a N-terminal RNase-like NYN (Nedd4-BP1, YacP Nucleases) domain, followed by two general RRM (RNA recognition motif), and a large C-terminal domain that is composed of 8 tandem repeats of newly identified OST-HTH/LOTUS (Oskar, Tudors-HTH/Limkain B1, Oskar, Tudors) structure^[9]^. It is proposed that the function of MARF1 in the control of oocyte meiotic resumption is probably elicited by its *N*-terminal RNase-like NYN domain through maintaining the RNA homeostasis within the oocytes, particularly the steady-state levels of *Ppp2cb*, the catalytic subunit of protein phosphatase 2A, PP2A^[89]^. The OST/LOTUS domain also exists in Drosophila Oskar and mammalian protein TDRD5/7 (Tudor domain containing proteins 5 and 7), which is predicted to bind with double stranded RNAs, especially those formed by small noncoding RNAs, such as piRNA, after hybridization with their targets^[13– 14]^. Oskar and TDRD5/7 play an important role in Drosophila germ cell fate specification and mouse male germ cell meiosis and retrotransposon silencing, respectively^[15– 17]^. Based on the structural and functional analogy, MARF1 is predicted to be a female counterpart of the male germ cellnuage components, such as PIWIs and TDRDs, and plays an essential role in the control of oocyte meiosis and genome integrity^[89]^. However, the exact function of MARF1 OST/LOTUS domain remains unknown.

Here, by knockin of eGFP at the *C*-terminus of MARF1, we created the transgenic line of mice that expressed the MARF1-eGFP fusion protein in the oocytes. However, the fusion protein was only detected by Western blot under the denaturing condition, no eGFP signals could be detected when the proteins were still in their folded confirmation in the live cells or fixed tissue. This suggests that the eGFP peptides probably undergo the similar conformational changes as MARF1 and are folded together with MARF1 during the post-translational processing and modification processes, thus getting masked in the native form. Adding a stretch of eGFP peptide at the C-terminus of MARF1 most likely compromised its normal function of the C-terminal domain because the *Marf1-eGFP*-KI females were infertile. The similar scenario had been observed when the Cre sequences were knocked into the *Amhr2* gene, which caused the complete loss of the function of AMHR2^[18]^. However, unlike the *Marf1* gene-trap KO female mice that display oocyte meiotic arrest, the *Marf1-eGFP*-KI oocytes could resume meiosis. This suggests that the function of MARF1, particularly that of the NYN-domain, is not completely lost in the *Marf1-eGFP*-KI oocytes. Therefore, the defects in meiotic progression and completion of the first meiosis observed in the *Marf1-eGFP*-KI oocytes indicate that it is most likely the MARF1's LOTUS domain that is responsible for the control of oocyte meiotic progression. This unexpected finding paves the path to further exploration of the function of this unique domain.

MARF1 is robustly expressed in oocytes. It is expressed in the oocytes at all the developmental stages starting from the primordial-follicle stage until after fertilization^[9]^. The maximal levels of expression are reached at the fully-grown stage oocytes, and are maintained up to the ovulated MⅡ-stage. The expression disappears rapidly once the oocytes are fertilized and form zygotes^[9]^. The observations made here in the present study that MARF1 is essential for the completion of the first meiosis and the formation of 2-cell stage embryos after fertilization is consistent with stably high levels of MARF1 expression during oocyte maturation and in MⅡ oocytes. These data thus collectively support the view that MARF1 is a key maternal factor essential for supporting oocyte meiotic maturation and early preimplantation development.

Orderly progression of meiosis in oocytes is driven by waves of activation and inactivation of the master cell cycle regulator, maturation-promoting factor, MPF^[19– 21]^. The phosphorylation status of the catalytic subunit, CDK1, and the steady-stage levels of the regulatory subunit, Cyclin B1, determine the activity of MPF^[19– 21]^. We observed here that GVB was delayed, and the extrusion of PB1 was nevertheless sped up in the *Marf1-eGFP*-KI oocytes, suggesting that the activation and inactivation of MPF was probably skewed. Previously, we showed that in the *Marf1* gene-trap KO oocytes, MPF was unable to be activated due to the upregulation of protein phosphatase 2A, PP2A, which kept MPF inactive. However, PP2A was not detected to be higher in the *Marf1-eGFP*-KI oocytes in the present study (data not shown), thus suggesting that MPF was kept low in the *Marf1-eGFP*-KI oocytes via different mechanisms, possibly through the degradation of Cyclin B1. Whether or not this defective progression of meiosis in *Marf1-eGFP*-KI oocytes is due to the lower levels of Cyclin B1 and hence lower levels of MPF activity in oocytes just before the onset of GVB or at the end of MI-to-AI transition warrants further investigation.

The establishment of bi-polar spindles, stable attachment of spindle microtubules to kinetochores, and the correct alignment of chromosomes at the spindles equators during the protracted prometaphase are all crucial prerequisite events for the orderly progression and accurate separation of homologous chromosomes during the first meiosis^[22]^. Errors in these processes are associated frequently with the formation of aneuploidy eggs^[3, 23]^. We found here that, associated with the accelerated extrusion of PB1, the incidences of homologous non-disjunction, chromosome misalignment, and aneuploidy were all increased in the *Marf1-eGFP*-KI oocytes. These data thus suggest that some spindle microtubule and chromosome dynamics-related evens must have gone away in the *Marf1-eGFP*-KI oocytes during the meiotic progression, and MARF1, particularly its C-terminal domain, participates in the control of these events. These data also imply that MARF1 may have specialized localization (e.g., colocalization with meiotic spindles and enrichment at the spindle poles) and interaction with some related cell cycle regulators during oocyte meiotic maturation, which are essential for the normal progression of oocyte meiosis. More studies are required to test these speculations.

Taken together, by studying with the serendipitously created *Marf1-eGFP*-KI mice, in which the function of the C-terminal domain of the expressed MARF1 protein was compromised, we revealed the unexpected role of MARF1 in the control of oocyte meiotic progression. Given that aneuploidy presents a growing reproductive health risk with the demographic tendency for an increased age of childbearing, we have much more to find out about the pathways that regulate the accurate segregation of homologous chromosomes during the oocyte meiotic maturation. The new role of MARF1 observed here will certainly be helpful in aiding exploration of the molecular control of oocyte meiosis.
